# A Complete SMOCkery: Daily Online Testing Did Not Boost College Performance

**DOI:** 10.1007/s10648-020-09588-0

**Published:** 2021-01-06

**Authors:** Daniel H. Robinson

**Affiliations:** College of Education, The University of Texas at Arlington, 504 Hammond Hall, Arlington, TX 76019 USA

**Keywords:** SMOC, internal validity, selection bias, quackery, differential attrition

## Abstract

In an article published in an open-access journal, (Pennebaker et al. *PLoS One, 8*(11), e79774, 2013) reported that an innovative computer-based system that included daily online testing resulted in better student performance in other concurrent courses and a reduction in achievement gaps between lower and upper middle-class students. This article has had high impact, not only in terms of citations, but it also launched a multimillion-dollar university project and numerous synchronous massive online courses (SMOCs). In this study, I present a closer look at the data used in the Pennebaker et al. study. As in many cases of false claims, threats to internal validity were not adequately addressed. Student performance increases in other courses can be explained entirely by selection bias, whereas achievement gap reductions may be explained by differential attrition. It is hoped that the findings reported in this paper will inform future decisions regarding SMOC courses. More importantly, our field needs watchdogs who expose such unsupported extravagant claims—especially those appearing in pay-to-publish journals.

When it comes to improving student achievement, there is no limit to novel interventions, treatments, and policies appearing in empirical, scientific journals. Unfortunately, fewer and fewer of these recommendations are based on experimental methods where the researcher randomly assigns students to treatment groups. In educational research journals, for example, the trends indicate fewer intervention studies and more observational studies accompanied with recommendations for practice (Hsieh et al. 2005; Reinhart et al. 2013; Robinson et al. 2007). Moreover, these recommendations for practice based on shaky evidence are often repeated in later articles (Shaw et al. 2010).

Fortunately, most of the snake oil recommendations for improving education do not make it to classrooms where they could actually do some damage. However, there are notable exceptions where such educational quackery has been implemented and continued to damage the already fragile reputation of educational research (e.g., Robinson and Bligh 2019; Robinson and Levin 2019). In a highly cited article (117 in Google Scholar and 45 in Web of Science as of November 25, 2020), Pennebaker et al. (2013) reported that an innovative online testing system resulted in better student performance in other courses and a reduction in achievement gaps between lower and upper middle-class students. This system is part of the first synchronous massive online course (SMOC) that was launched in 2013 at the University of Texas at Austin. News of the impressive results by Pennebaker et al. spread quickly and were subsequently cited in several other articles. For example, Takooshian et al. (2016) stated:

An especially encouraging result was reported by University of Texas researchers who compared the effectiveness of an online version of introductory psychology with a traditional version (Pennebaker et al. 2013). Not only did psychology exam scores increase by approximately half a letter grade when the course was taught online—the socioeconomic achievement gap in course grades was cut in half. (p. 142)

Similarly, Straumsheim (2013) interpreted the results as follows:

As more and more of the coursework continued to shift toward digital, the data showed a clear trend: Not only were students in the online section performing the equivalent of half a letter grade better than those physically in attendance, but taking the class online also slashed the achievement gap between upper, middle and lower-middle class students in half, from about one letter grade to less than half of a letter grade…“We are changing the way students are approaching the class and the way they study,” Pennebaker said…“That’s one thing that I’m actually most excited about…This project could never have been built here at the university without heavy research behind it.”

Originally, the professors hoped the class would attract 10,000 non-university students willing to pay a few hundred dollars for the for-credit class. Indeed, the headline for a *Wall Street Journal* article about the pair’s innovation trumpeted “Online class aims to earn millions.” That hasn’t happened. The class, offered each fall, still mostly consists of regular University of Texas undergrads. And while Gosling believes the model will eventually spread to other universities, as far as he knows it hasn’t done so yet, perhaps because of the expertise and hefty investment required. Still, the model has been so successful the university has since developed SMOC versions of American government and U.S. foreign policy classes (Clay 2015, p. 54).

Despite the “hefty investment” required, based on the fantastic findings and press from the Pennebaker et al. (2013) study, in 2016, the University of Texas at Austin named Pennebaker the executive director of Project 2021 that was supposed to “revamp undergraduate education by producing more online classes” (Dunning 2019, p. 1). The university initially committed $16 million to Project 2021, which included monies for increasing the number of SMOC production studios.

The first piece of the grand idea came from Professor James W. Pennebaker. He and a colleague had brought software into a class that allowed professors to quiz students during every class, and the data showed that learning disparities between students decreased. They then created an online course, initially livestreamed from a studio using greenscreens. It was called a “synchronous massive online course,” or SMOC, and UT was proud that it was the first. (Conway 2019, p. 1)

Pennebaker had also recently been awarded the APA Award for Distinguished Scientific Applications of Psychology. This seemed to be a perfect example of a distinguished scientist applying findings from psychology to improve undergraduate education—something that is unfortunately rare (Dempster 1988). Also, unfortunately, things did not turn out so well. By 2018, after only two years into a five-year initiative, Project 2021 was suddenly dead and the controversy surrounding it was covered in the *Chronicle of Higher Education* (Ellis 2019).

It seems the initiative didn’t grow from student demand or from research showing a definitive opportunity to serve students better. It grew from one professor who had a success in one course, and from the outside momentum in the education world toward digitized or “reimagined” learning experiences—of which data about learning outcomes is actually pretty shaky. (Conway 2019, p. 1)

Indeed, the data are “shaky.” The evidence used to support the SMOC was not based on a carefully controlled comparison between the SMOC and face-to-face courses. Instead, the Pennebaker et al. (2013) study was an ex post facto comparison of students who took a completely in-class version of the introductory psychology course in 2008 with those who had the online quizzes in 2011. As mentioned earlier, this “observational” approach is consistent with the latest trends in educational research where researchers avoid random assignment of students to experimental conditions. Despite the shaky evidence, the SMOC did not die along with Project 2021. On the contrary, the production of SMOC courses at the University of Texas was ramped up. Compared to the 26 that were produced in the 2015–2016 academic session, 90 were produced during 2018–2019, and over 29 were planned for Summer 2019 (Dunning 2019).

How could one article have such an impact? How was the University of Texas at Austin duped into spending time and money on this bullsh-initiative? Undoubtedly, the extraordinary claims had something to do with it. The notion that a single course could have a causal effect of improving student performance in other courses both the following semester and, incredulously, the *same* semester is simply amazing. The other claim of reducing achievement gaps likely resonated with most educators who have been working on this problem for decades. But, similar to the first claim, there are no known interventions that reduce achievement gaps. Otherwise, we would be using them and would no longer have gaps.

## Method

In this study, I examined these claims by taking a closer look at the data used in the Pennebaker et al. (2013) article. As previously mentioned, threats to internal validity were not adequately addressed. Thus, I simply looked at alternative reasons why the daily online testing students in 2011 experienced advantages over the traditional instruction students from three years earlier (2008).

## Results

As with any comparison study that does not randomly assign students to experimental conditions, one should first look for possible preexisting student differences that could explain any subsequent performance differences. The first possible threat to internal validity I examined was history. In other words, was there something that occurred between 2008 and 2011 that could explain the increase in GPA in the other courses? Grade inflation is certainly a possibility that could account for some of the improved performance of students in 2011 compared with those in 2008. Indeed, the University of Texas at Austin undergraduate average GPA had risen steadily since a few years before 2008 and a few years after 2011.

The actual difference between undergraduate average GPA in 2011 compared with 2008 is 0.07 (3.27 − 3.20). This difference, however, is considerably less than the differences in GPAs reported by Pennebaker et al. (2013) of 0.11 and 0.12. Thus, although grade inflation could partly explain the GPA differences between the 2008 and 2011 students, it cannot fully account for the differences.

The next possible threat to internal validity I examined was selection bias. As many people know, there exist, at most universities, differences in GPA among various majors. For example, it is well known that education majors typically have higher GPAs than do engineering majors. Thus, if one of the groups in a comparison study has more students from an “easier” or “harder” major than the other group, this preexisting difference could surface in any outcome variables that use the same measure or a similar one. In the Pennebaker et al. (2013) study, indeed, they used student semester GPA as the main outcome measure to gauge whether the daily online testing led to better student performance in their other courses.

Now, the assumption here is that students typically take most of their courses in their major area. In fact, at the University of Texas at Austin, students take only 42 h (out of 120 total) of core courses. The rest are in their major or minor areas and a handful of electives. Thus, if one assumes that students take most of their courses in their major or closely related areas, then it can also be assumed that their GPA for any given semester will reflect group differences that exist according to major. In other words, grades in social work courses are typically higher than those in natural science courses. Thus, we would expect a group that has more social work students to have a higher GPA than a group with fewer such students. The opposite would be true for a group with more business students.

I accessed the student major data for the 994 students who were enrolled in the introductory psychology course at the University of Texas at Austin in the Fall semester of 2008 and for the 941 enrolled in 2011. Note that these totals are different from the 935 and 901, respectively, that were reported in Pennebaker et al. (2013). Table 1 below shows the average GPAs for all courses by subject areas by year (2008 and 2011), and the numbers of students in the psychology courses who were majoring in those areas.

**Table 1:** GPAs by major for both the 2008 and 2011 groups. Particular differences are highlighted in *italic* typeface

2008				2011		
Major	*N*	GPA	Weighted	*N*	GPA	Weighted
Business	108	3.30	356.4	92	3.29	302.68
Education	*56*	*3.63*	203.28	*93*	*3.59*	333.87
Engineering	*84*	*3.18*	267.12	*41*	*3.27*	134.07
Fine arts	29	3.51	101.79	19	3.56	67.64
Communication	59	3.37	198.83	38	3.33	126.54
Natural sciences	*304*	*2.92*	887.68	*263*	*3.08*	810.04
Geosciences	0			4	2.77	11.08
Liberal arts	*313*	*3.18*	995.34	*216*	*3.22*	695.52
Nursing	10	3.80	38	21	3.84	80.64
Social work	31	3.70	114.7	23	3.59	82.57
Undergrad studies	0			131	*3.44*	450.64
Totals	994	*3.18*	3163.14	941	*3.29*	3095.29

To get the expected GPA of the entire class simply based on student major, I multiplied the number of students by the average GPA of the subject area courses to get a weighted number. I then summed the weighted numbers and divided by the total number of students to get a weighted average GPA for each group. This “major” effect size for the online testing group over the traditional group (3.29 − 3.18 = 0.11) is almost *identical* to the reported advantages reported by Pennebaker et al. (2013) for both the concurrent semester (3.07 − 2.96 = 0.11) and the subsequent semester (3.10 − 2.98 = 0.12). Thus, the student performance increases can be fully explained by selection bias: there were different proportions of students from majors that naturally tend to have higher or lower grades in those major courses. With regard to internal validity, when an alternative explanation exists that can account for an “experimental” effect, then that experimental effect becomes bogus.

Finally, as for the reduction in achievement gaps, Pennebaker et al. (2013) acknowledged that the online testing courses were more rigorous due to daily quizzes. Typically, with increased rigor comes increased drop rates. I decided to examine a third threat to internal validity, differential attrition, that might explain the reduction in achievement gaps. Differential attrition occurs when participants in one group drop out of the study at a higher rate than other groups. For example, suppose a company that runs a fitness bootcamp claims that its average participant loses 15 pounds by the end of the four-week camp. However, out of every 100 participants that show up on day one, an average of 80 fail to finish the entire bootcamp due to its extreme rigor. Of the 100 people in the control group who did not participate, zero drop out (no rigor) and thus remain at the end of the four weeks. Weight loss comparisons are made between the 20 who finished the bootcamp and the 100 control group participants. Thus, the bootcamp’s claim is exaggerated. Whereas the completers might experience an impressive weight loss, the average person who pays for the camp might not experience any weight loss.

Similarly, in 2008 when the psychology course was less rigorous with no daily quizzes, only 32 students dropped the course. Comparatively, in 2011 when the rigor was increased, almost twice as many students (58) dropped. Students from lower SES families unfortunately tend to drop courses at higher rates than do their richer counterparts. It is certainly possible that many of these students who dropped were from the low middle class. Thus, any analysis would show a reduction in the performance differences between the low and high middle-class students. This certainly is not as much of a “smoking gun” as the selection bias findings. But does anyone actually believe that daily online testing would reduce achievement gaps?

## Discussion

During the current pandemic in 2020, many colleges and universities are struggling to deliver online instruction. Scholars and practitioners are arguing whether online instruction is just as effective as face-to-face instruction. The encouraging findings reported by Pennebaker et al. (2013) not only allowed some to conclude that online instruction may be equally effective, but the suggestion that online may be *more* effective than face-to-face undoubtedly spurred efforts to shift more and more instruction to online environments. But, as the present findings suggest, such enthusiasm for online instruction may not be supported by the data.

Are there any negative consequences of assuming that a SMOC version of a course might be better than a face-to-face version? How many more SMOCs should the University of Texas at Austin develop? Daily testing benefits are a robust phenomenon in cognitive psychology (e.g., Roediger and Karpicke 2006) and no reasonable person would argue against employing this strategy in any course. However, the benefit of having students frequently retrieve newly learned information is only revealed during later comprehensive testing such as a final exam. No one has ever claimed that frequent testing can improve student performance in other courses. And there are certainly no course-wide interventions that improve student performance in other concurrent courses! Such unicorns have yet to be found. Similarly, reducing achievement gaps has been a goal in education for over 50 years—ever since the Elementary and Secondary Education Act of 1965. Sadly, very little progress has been made on this front. Daily online testing is no magic bullet that will solve the problem.

This is certainly not the first time that findings published a widely cited educational research article have been later refuted. Recently, Urry et al. (in press) conducted a direct replication of Mueller and Oppenheimer (2014) who had found that taking notes using a laptop was worse for learning than taking notes by hand. The findings of Urry et al. refuted the earlier claim, but not until the Muehler and Oppenheimer study had been cited 278 times (Web of Science, as of November 25, 2020)!

In the early stages of the pandemic in 2020, US President Trump promoted the drug hydroxychloroquine as an effective treatment of Covid-19. Unfortunately, there was then, and remains today, absolutely no evidence that the drug improves outcomes for those inflicted with Covid-19 (Jha 2020). In fact, some studies have shown that it causes more harm than good. Yet, many Americans began taking the drug. This is understandable, given that so many people have a hard time with the notion of scientific evidence. But can we as easily excuse public research universities from making similar mistakes? Year after year, with the arrivals of newly appointed provosts and presidents, universities tout their latest bullsh-initiatives that will cost millions of dollars and promise to be game changers. Does anyone ever follow up to see if such spending did any good? Should universities appoint watchdogs to ensure that money is not wasted chasing such windmills?

Finally, what about the responsibilities of the scientific community? As previously mentioned, the Pennebaker et al. (2013) study has been cited in several scientific publications according to the Web of Science. How did it first get past an editor and reviewers? *PLOS ONE* claims to be a peer-reviewed open-access scientific journal. From their website, they claim to “evaluate research on scientific validity, strong methodology, and high ethical standards.” They also report that the average time to the first editorial decision for any submitted paper is 12–14 days. Most reputable journals take much longer than this. During my time as an associate editor for the *Journal of Educational Psychology*, I handled over 500 submissions. The average number of days to the first editorial decision was over 30 days. The fact that *PLOS ONE* is much faster may reflect a difference in the review process and the $1700 publication fee.

It is hoped that future incredulous findings will be fully vetted during the review process before appearing in widely available outlets. Perhaps authors should not be encouraged to publish their work in strictly pay-to-publish journals. All members of the scientific community need to consider using the strongest possible methods and carefully note study limitations. Pennebaker et al. (2013) could have easily designed a randomized experiment to test the effectiveness of the SMOCs. With almost one thousand students enrolling in the introductory psychology course each semester, it would have been easy to randomly assign half of them to either a SMOC or control, face-to-face section. Finally, we should all take care to only cite studies that have scientific merit and not repeat bogus claims. If bogus claims do find their way into journals, we have a duty to call out such claims.
